# Folic acid, minerals, amino-acids, fatty acids and volatile compounds of green and red lentils. Folic acid content optimization in wheat-lentils composite flours

**DOI:** 10.1186/s13065-018-0456-8

**Published:** 2018-08-04

**Authors:** Adriana Paucean, Ovidiu P. Moldovan, Vlad Mureșan, Sonia A. Socaci, Francisc V. Dulf, Ersilia Alexa, Simona M. Man, Andruţa E. Mureșan, Sevastița Muste

**Affiliations:** 1 Faculty of Food Science and Technology, University of Agricultural Sciences and Veterinary Medicine Cluj-Napoca, 3-5 Calea Mănăștur Street, 400372 Cluj-Napoca, Romania; 2 Faculty of Agriculture, University of Agricultural Sciences and Veterinary Medicine Cluj-Napoca, 3-5 Calea Mănăștur Street, 400372 Cluj-Napoca, Romania; 3 Banat’s University of Agricultural Sciences and Veterinary Medicine “King Michael I of Romania” from Timisoara, 119 Calea Aradului, 300645 Timișoara, Romania

**Keywords:** Folic acid, Lentil, Wheat, Composite flours, Breadmaking technology

## Abstract

The advanced biochemical characterisation of green, red lentil and wheat flours was performed by assessing their folic acid content as well as individual minerals, amino acids, fatty acids and volatile compounds. Moreover, a nutritionally improved wheat–lentil composite flour, with a content of 133.33 μg of folic acid/100 g, was proposed in order to assure the folic acid daily intake (200 μg) for an adult person. The wheat and lentil flours percentages used for the composite were calculated by using the equations for total material balance and folic acid content material balance. Bread was selected as model food for the composite flour due to its high daily intake (~ 250 g day^−1^) and to its great potential in biofortification. By this algorithm, two composite flours were developed, wheat–green lentil flour (22.21–77.79%) and wheat–red lentil flour (42.62–57.38%), their advanced biochemical characteristics being predicted based on the determined compositions of their constituents. The baking behaviour of the new developed wheat-lentils composite flours with optimised folic acid content was tested. In order to objectively compare the bread samples, texture profile analysis was considered the most relevant test. A good baking behaviour was observed for the wheat–red lentil bread, while for the wheat–green lentil composite flour, encouraging results were obtained.

## Introduction

Nowadays, a great interest in the production and use of lentil (*Lens culinaris*) in food formulation and preparation has been noticed due to their high nutritional value. Lentil serves as a good source of carbohydrates (e.g., fiber, resistant starch and oligosaccharides), proteins, vitamins and minerals. Lentil has an excellent macro and micronutrient profile and favorable levels of mineral bioavailability enhancing factors [1]. Due to their high content of amino acids such as lysine and arginine, lentil could complement cereal proteins improving the overall nutritional value of the food [2]. In addition to providing essential and non-essential amino acids and carbon skeletons for the metabolic needs of the human body, lentils are sources of lectins and protease inhibitors that, in the light of the latest research, are described as biologically active proteins [3]. Lentils have relatively low fat content, the fatty fractions being saturated fatty acids (SFA), 16.7%; monounsaturated fatty acids (MUFA), 23.7% and polyunsaturated fatty acids (PUFA), 58.8% [4]. Also, they are considered as a potential whole food source for people affected by micronutrient malnutrition [5, 6]. The mineral elements of lentils include Fe, Zn, Cu, Mn, Mo, while Mg, P, Ca and S are present in relatively high levels. In addition, lentils have a low Na and relatively high K contents, with a K:Na ratio of about 30:1–90:1 [3]. Bioactive compounds of lentils include polyphenols (flavonols, tannins, phenolic compounds), phytate, phytosterols, minerals, vitamins, oligosaccharides, resistant starch, proteins, bioactive peptides and saponins are responsible for health improving effects. The scientific literature emphasizes the beneficial effects of lentils consumption for the cardiovascular diseases, diabetes, the body weight control, several types of cancers [6].

Lentils are a significant dietary source of vitamins including folate, thiamin (B1) and riboflavin (B2), niacin, pantothenic acid and pyridoxine. Folate (vitamin B9) has a central role in fundamental cell processes, such as nucleic acid and amino acid biosynthesis, while insufficient folate intake may lead to folate deficiency disease megaloblastic anaemia and increased risks for neural vessels defects, as well as other malformations [7]. Other studies reported that folates play important roles in the aetiology of cardiovascular diseases and different types of cancer [8]. The main folates dietary sources of folates are liver, fresh dark leafy vegetables, legumes (e.g., lentils, cowpeas, chickpeas), wheat germ and yeast [9].

The increasing interest in healthy eating over the last two decades determined the development of a range of new functional foods. Due to their usage in various food matrices, plant ingredients can be consumed increasingly as they have many health benefits [10]. Grain legumes have a high protein and fiber content, they are gluten-free, have a low glycemic index, an antioxidant potential and numerous functional properties such as water binding capacity and fat absorption. These make grain legumes very useful as novel ingredients to improve the nutritional quality of foods [11]. Therefore, interest in their consumption seems to be increasing over the world.

Cereals are the most important group of food crops produced in the world and they constitute the raw commodities of the bakery products. Cereal contribution to the human diet is of major importance since the yearly consumption per capita is 147–150 kg per person. According to World Health Organization [12], several European countries recommend a daily bread intake of about 250 g, which corresponds to 4–8 slices depending on national food habits. In this context bread as a staple food could be a potential product for biofortification [13].

Therefore, this study aimed (1) to assess the folic acid content, the minerals, amino acids, fatty acids composition as well as the volatile compounds of wheat, green and red lentils flours and (2) to develop and characterize nutritionally improved wheat–lentil composite flours able to assure the folic acid daily intake for an adult person. Previous studies have been focused on determining lentils compositional properties and their role in human health and nutrition [2, 14–17] but to the best of our knowledge, there is a lack of information regarding the existence as well as the advanced biochemical characterisation of any wheat–lentil composite flours with an optimised folic acid content.

## Materials and methods

### Materials

Two varieties of lentils (*L. culinaris*), categorised based on their colours, red (*Imperial*) and green (*Laird*), were purchased from a specialized local store being processed by grinding to a fine flour (< 300 μm) on a Grindomix (GM200) laboratory mill at 10,000 rot min^−1^ for 50 s. The obtained flours were codified as following: green lentil flour (FLG) and red lentil flous (FLR). Wheat (*Arieşan* variety) flour (WF) sample was produced by a local mill, Boromir, and sold as type 650 according to ash content by Romanian classification.

### Determination of folic acid

#### Standard solution

Folic acid standard Supelco from Sigma-Aldrich-Germany was used. Folic acid stock standard solution was prepared by dissolution of 1 mg folic acid in 1 mL 0.1N NaOH. Aliquots of this solution were taken to prepare analytical solutions in phosphate pH 7.0 buffers, and used to calibrate an HPLC UV detector [18].

#### Extraction

An amount of 0.5 g sample was extracted with 5 mL of phosphate buffer pH 7.0 (0.25 mol L^−1^ dibasic sodium phosphate and 0.37 mol L^−1^ monobasic potassium phosphate). The mixture was shaken for 30 min in a rotational shaker, and centrifuged at 3000 rpm for 15 min. The supernatant was filtered through a 0.45 µm nylon membrane (Sigma-Aldrich) and 20 µL extract was injected manually.

#### Chromatography conditions

Determination of folic acid was carried out using an high-performance-liquid chromatography (HPLC) Agilent 1200 system from Agilent Technologies (USA), equipped with: solvents degasser, quaternary pump, UV–VIS detector, computer software, column thermostat, manual injector and Lichrospher 100 RP-18, (250 × 4.6 mm, 5 µm) column (Agilent Technologies, USA). The column temperature was maintained at 25 °C. Gradient elution with acetonitrile and acetic acid 1% (20/80 v/v, pH 2.8), isocratic system, was used to separate folic acid. The flow rate was 0.5 mL min^−1^. Folic acid was detected by a UV detector at a wavelength of 280 nm [18]. For quantitative determination of folic acid peak areas of the sample, chromatograms were correlated with the concentrations according to the calibration curve (y = 131.86x + 64).

### Analysis of macro and microelements

Macro and microelements were determined by atomic absorption spectrophotometry. An amount of 3 g were burned 10 h at 550 °C in furnace (Nabertherm B150, Lilienthal, Germany). The ash was dissolved in HCl 20% and was transferred by a final volume of 20 mL in a volumetric flask. The macroelements (K, Ca, Mg) and microelements (Fe, Cu, Zn and Mn) were determined by AAS (Varian 220 FAA equipment). Mix standard solutions (ICP Multielement Standard solution IV CertiPUR) were purchased from Merck. All chemicals and solvents used in this study were of analytical grades. The results were expressed as related to the fresh weight basis. Each value is the mean of three (n = 3) independent determinations.

### Analysis of amino acids

Sample sizes of 0.5 g were hydrolysed in 10 mL 6-N hydrochloric acid for 24 h at 110 °C. The sample was filtered through the filter Millipore 0.2 Pm, diluted sample compared 1:10 with HCl 0.1 N and injected into the chromatograph. Chromatographic conditions: Column chromatography AMINOPAC PA10 (2 × 250 mm, P/N 055406), Precolumn AMINOPAC PA10 (2 × 50 mm, P/N 055407), gradient: water/NaOH 250 mM/sodium acetate 1 M, flow rate of mobile phase: 0.25 mL min^−1^, Reference electrode: pH/Ag/AgCl, temperature of column 30 °C. Sulphur-containing amino acids and tryptophan were not analysed.

### Determination of fatty acid composition

#### Extraction of lipids

The total lipids were extracted from 10 g aliquots by using chloroform: methanol mixture [19]. The recovered lipids were weighed and transferred to vials with 4 mL of chloroform and stored at − 18 °C for further analysis.

#### GC–MS analysis of FAMEs

The total lipids were transesterified into fatty acid methyl esters (FAMEs) using the acid-catalyzed method [20]. The separation, identification and quantitation of the FAMEs were carried out by gas chromatography–mass spectrometry (GC–MS), using a PerkinElmer Clarus 600 T GC–MS (PerkinElmer, Inc., Shelton, CT, USA) [21]. The samples (1 μL) were injected into a Supelcowax 10 (60 m × 0.25 mm i.d., 0.25 μm film thickness; Supelco Inc., Bellefonte, PA, USA) capillary column in the split injection mode (split ratio 1:24). Helium was used as carrier gas with a flow rate of 0.8 mL min^−1^. The GC program was as follows: initial temperature, 140 °C; increase by 7 °C min^−1^ to 220 °C; and hold for 23 min. The injector temperature was set at 210 °C. The mass spectra were recorded in the positive-ion mode at 70 eV and a trap current of 100 μA with a source temperature of 150 °C. The mass scans were performed within the range of m/z 22–395 (0.14 scan s^−1^ with an intermediate time of 0.02 s between the scans). FAMEs were identified by comparison of their retention times with those of the authentic standards (37 component FAME Mix, Supelco no. 47885-U) and the resulting mass spectra to those in the database (NIST MS Search 2.0). The amount of each fatty acid was calculated as peak area percentage of total fatty acids.

### Extraction and analysis of volatile compounds

The extraction of volatile compounds from 3 g of sample was performed using the in-tube extraction technique (ITEX) [22]. The analysis of volatile compounds was carried out on a GCMS QP-2010 (Shimadzu Scientific Instruments, Kyoto, Japan) model gas chromatograph–mass spectrometer equipped with a CombiPAL AOC-5000 auto sampler. The volatile compounds were separated on a Zebron ZB-5 ms capillary column of 30 m × 0.25 mm i.d and 0.25 mm film thickness. The carrier gas was helium, 1 mL min^−1^ and the split ratio 1:5. The temperature program used for the column oven was: 30 °C (held for 5 min) rising to 110 °C with 4 °C min^−1^ and then heated to 250 °C with 15 °C min^−1^ and held for 5 min. The injector, ion-source and interface temperatures were set at 250 °C. The MS mode was electron impact (EI) at ionization energy of 70 eV. The mass range scanned was 40–400 m z^−1^. The volatile compounds were tentatively identified based on the spectra of reference compounds from NIST27 and NIST147 mass spectra libraries and verified by comparison with retention indices drawn from http://www.pherobase.com or http://www.flavornet.org (for columns with a similar stationary phase to the ZB-5 ms column). All peaks found in at least two of the three total ion chromatograms (TIC) were taken into account when calculating the total area of peaks (100%) and the relative areas of the volatile compounds.

### Bread samples formulation

A straight dough method for bread samples preparation and the following formula (for control bread) was used: wheat flour 100%, dried yeast 2%, salt 2% (amount of ingredients in reference to flour) and water needed for preparation of dough with farinograph consistency of 500 BU. In the case of samples prepared with GL/RL, blends of W–GL: 22.21–77.79% and W–RL: 42.62–57.38% were used as substitute of 100% WF.

Dough was kneaded using a single spiral mixer (type Hobart) for 12 min; dough with 24 °C temperature was divided into pieces of 1000 g and the following steps were used: rounding, first pre-proofing (20 min, 25 °C, relative humidity (RH) 60%), second rounding, second pre-proofing (30 min, 25 °C, 60% RH), final shaping, final proofing (70 min, 30 °C, 80% RH), baking in electrical oven (40 min, 225 °C, Zanolli type), cooled and subjected to analysis.

### Texture profile analysis for bread samples

CT 3 Texture Analyzer (Brookfield Engineering Labs), equipped with 10 kg load cell and the TA11/1000 cylindrical probe (25.4 mm diameter AOAC Standard Clear Acrylic 21 g, 35 mm length) was used in a texture profile analysis test (40% target deformation, 1 mm s^−1^ test and post-test speed, 5 g trigger load, and 5 s recovery time). The specific texture parameters were computed by Texture Pro CT V1.6 software.

## Results and discussion

### Folic acid content

Both varieties of lentils, green and red, show high amounts of folic acid, with highest content in red lentil flour (2244.8 µg kg^−1^) (Table 1). Our results are consistent with those obtained by different analytical methods, such as LC–MS method [23] and LC–FD method [24]. The WF content in folic acid was much smaller than that in lentils flours but according with the folic acid content of unfortified wheat flour. In some countries folic acid fortification has been mandatory for cereal products that have resulted in a significant increase in the mean folate intake. However, in Europe mandatory fortification has not yet been taken, in several Western European countries the recommended folic acid daily intake is ranging between 200 and 400 μg (adults and pregnant women) as was reported by different studies which have been dealing with the folate intake of the population [8, 25]. The US Food and Drug Administration (FDA) implemented a program to fortify all flour and cereal products with folic acid at levels of 1400 μg kg^−1^ of product [25].

**Table 1:** Folic acid, minerals, amino acids and fatty acids content in wheat flour (WF), green lentil flour (FLG) and red lentil flour (FLR)

Chemical compounds	WF	FLG	FLR
Folic acid (µg/100 g)	10.62 ± 0.1	168.36 ± 2.3	224.48 ± 2.6
Minerals (mg/100 g DM)			
Calcium (Ca)	15.12 ± 0.1	77 ± 0.6	89.11 ± 1.6
Iron (Fe)	1.11 ± 0.14	7.55 ± 0.1	8.55 ± 0.8
Potassium (K)	101 ± 0.09	955 ± 1.5	1055 ± 2.5
Zinc (Zn)	0.85 ± 0.1	4.78 ± 0.9	4.38 ± 1.2
Magnesium (Mg)	26.01 ± 0.4	122.21 ± 1.12	127.12 ± 1.4
Manganese (Mn)	0.84 ± 0.2	1.63 ± 0.2	1.83 ± 0.08
Copper (Cu)	0.18 ± 0.03	1.31 ± 0.2	1.24 ± 0.5
Amino acids (g/16 g N)			
Lysine	0.263 ± 0.03	5.713 ± 0.18	5.861 ± 1.2
Threonine	0.272 ± 0.02	4.632 ± 0.1	4.258 ± 1.1
Valine	0.411 ± 0.01	4.132 ± 0.2	3.799 ± 1.4
Isoleucine	0.322 ± 0.02	3.131 ± 0.2	3.1 ± 0.8
Leucine	0.723 ± 0.03	6.321 ± 0.03	6.154 ± 0.9
Phenylalanine	0.519 ± 0.1	4.778 ± 0.1	4.287 ± 0.13
Arginine	0.393 ± 0.02	7.239 ± 0.1	7.723 ± 0.15
Alanine	0.329 ± 0.01	4.243 ± 0.2	3.943 ± 1.2
Proline	1.191 ± 0.01	4.641 ± 0.17	4.387 ± 0.2
Glycine	0.361 ± 0.04	4.167 ± 0.09	3.841 ± 0.9
Glutamic acid	3.451 ± 0.02	14.712 ± 1.9	14.767 ± 1.7
Aspartic acid	0.394 ± 0.01	12.643 ± 1.2	12.213 ± 1.09
Fatty acids (% of total fatty acids)			
10:00	0.02 ± 0.004	0.01 ± 0.001	0.01 ± 0.003
12:00	0.03 ± 0.005	0.02 ± 0.007	0.04 ± 0.009
14:00	0.14 ± 0.003	0.49 ± 0.1	0.42 ± 0.008
15:00	0.1 ± 0.001	0.12 ± 0.004	0.17 ± 0.041
Aza	0.09 ± 0.005	0.03 ± 0.001	0.03 ± 0.006
16:00	18.95 ± 1.05	16.43 ± 0.95	14.11 ± 0.39
16:1(n − 9)	0.16 ± 0.004	0.07 ± 0.002	0.13 ± 0.071
16:1(n − 7)	0.13 ± 0.02	0.2 ± 0.009	0.07 ± 0.004
17:00	0.08 ± 0.003	0.23 ± 0.007	0.2 ± 0.061
17:1(n − 9)	0.04 ± 0.001	0.09 ± 0.003	0.11 ± 0.002
18:00	1.4 ± 0.021	2.47 ± 0.06	1.57 ± 0.25
18:1(n − 9)	13.03 ± 1.16	22.44 ± 0.99	29.84 ± 1.40
18:1(n − 7)	0.87 ± 0.32	0.89 ± 0.041	0.71 ± 0.002
18:2(n − 6)	59.13 ± 2.36	44.66 ± 1.31	39.23 ± 1.35
18:3(n − 3)	4.26 ± 0.90	9.53 ± 0.94	10.62 ± 0.71
20:00	0.18 ± 0.007	0.56 ± 0.07	0.56 ± 0.098
20:1(n − 9)	0.52 ± 0.048	0.5 ± 0.021	0.82 ± 0.024
20:2(n − 6)	0.08 ± 0.001	0.06 ± 0.001	0.08 ± 0.001
21:00	0.03 ± 0.002	0.11 ± 0.004	0.11 ± 0.07
22:00	0.34 ± 0.05	0.47 ± 0.011	0.48 ± 0.009
22:1(n − 9)	0.07 ± 0.011	0.11 ± 0.009	0.16 ± 0.033
24:0	0.23 ± 0.46	0.32 ± 0.013	0.34 ± 0.06
24:1(n − 9)	0.05 ± 0.001	0.03 ± 0.001	0.04 ± 0.002
ΣSFAs	21.59	21.39	18.17
ΣMUFAs	14.86	24.33	31.87
ΣPUFAs	63.46	54.26	49.93
n − 3ΣPUFAs	4.26	9.53	10.62
n − 6ΣPUFAs	59.21	44.73	39.31
n − 6/n − 3	13.91	4.69	3.7
PUFAs/SFAs	2.94	2.54	2.75
Total fat (%dw)	0.7	1.5	1.6

Values are the means of three measurements (mean ± SD, n = 3)

*C10:0* capric, *C12:0* lauric, *C14:0* myristic, *C15:0* pentadecanoic, *C16:0* palmitic, *C16:1n − 9* cis-7 hexadecenoic, *C16:1n − 7* palmitoleic, *C17:0* margaric, *C18:0* stearic, *C18:1n − 9* oleic, *C18:1n − 7* vaccenic, *C18:2n − 6* linoleic, *C18:3n − 3* α-linolenic, *C20:0* arachidic, *C20:1n − 9* 11-eicosenoic, *C20:2n − 6* eicosadienoic, *C21:0* heneicosanoic, *C22:0* behenic, *C22:1n − 9* erucic, *C24:0* lignoceric, *C24:1n − 9* nervonic acids, *SFAs* saturated fatty acids, *MUFAs* monounsaturated fatty acids, *PUFAs* polyunsaturated fatty acids

### Macro and microelements content

Results showed that K was the most abundant element in lentil flours with values of about 9550 mg kg^−1^ for FLG and 10,550 mg kg^−1^ for FLR (Table 1). Also, FLG and FLR recorded important content of Mg and Ca, in the case of macronutrients and Fe, Cu, Zn as micronutrients. The mineral contents were similar to those reported for lentils varieties [14, 15]. Iron deficiency anaemia is the most common nutritional disorder in the world. Iron is an integral part of protein involved in oxygen transport (haemoglobin, myoglobin), energy metabolism (cytochromes) and steroid and xenobiotic metabolism [26]. Lentil is high in bioavailable form of Fe therefore it has great potential as a whole food source of bioavailable iron [1]. Zn deficiency is responsible for stunting, lower respiratory tract infections, malaria, and diarrhoeal disease. Lentils are attractive candidates for mineral biofortification, especially due to their low levels of phytic acid as have been reported for lentils grown in northern temperate climates [5].

### Amino acids content

Data presented in Table 1 shows the amino acid profile of WF, FLG, FLR. Our results for red and green lentils composition in amino acids are consistent with those reported by other studies [14, 16, 27].

Both FLG and FLR showed high amounts in all tested essential amino acids comparatively to WF. Lysine, isoleucine, leucine, phenylalanine, threonine and valine contents were found to be much higher than those from WF. Glutamic acid, aspartic acid and arginine were found to be major non-essential amino acids in the tested samples. Nutritive value of protein is determined by the pattern and quantity of essential amino acids present. The presence of one or more of the essential amino acids in adequate amounts would increase the nutritive value of the protein [14]. Therefore, lentils protein could very well complement the WF’s protein.

### Fatty acid composition

Data about the qualitative and quantitative composition of fatty acids for WF, red/green lentils are summarised in Table 1. A total of 23 fatty acids were identified in analysed samples by GC/MS-FID analysis. Fatty acid profile of FLG, FLR reveals that lentils lipids are a good source of the nutritionally essential linoleic and oleic acids. Linoleic acid (18:2(z,z)n − 6), palmitic acid (16:0) and oleic acid (18:1n − 9) were the dominating fatty acids of FLR, FLG. Also, α-linolenic acid (18:3n − 3) contents of FLR and FLG were highest than WF. Wheat flour showed the highest value of linoleic and palmitic acids than both varieties of lentils. PUFAs have several beneficial effects on cardiovascular disease including improved blood lipid profile, improved insulin sensitivity, lower incidence of type 2 diabetes and anti-arrhythmic effects [6, 17]. The PUFA n − 6/n − 3 ratio had a mean of 4.69 for red lentil and 3.7 for green lentil. A value of around 4, for n − 6/n − 3 PUFA ratio, was coupled with a 70% decrease in total mortality caused by cardiovascular disease [28]. Another study reported results for n − 6/n − 3 ratio in 20 lentils from different cultivars as ranged from 3.4 to 4.9 [14].

### Volatile compounds

The volatile fingerprints of wheat, red/green lentils flours were determined using the ITEX/GC–MS technique and a total of 9 compounds were identified in tested samples (Table 2). Using the above mentioned database or other literature sources [29, 30] the characteristic odour of each detected volatile compound is also specified. The volatile constituents found in the analysed samples include alcohols, aldehydes, as well as furans, ketones and other classes of compounds. The main volatile compound identified in lentils flours was limonene, with the highest content in red lentil flour (58.29%).

**Table 2:** Mean relative peak areas (expressed as % from total peak areas) and standard deviations of volatile compounds from wheat, red/green lentils flour samples analysed by HS-ITEX/GC–MS technique

Volatile compound	Odour perception	Samples		
		WF	FLG	FLR
1-Hexanol	Green, grass, fat	31.86 ± 0.24	5.93 ± 0.81	4.19 ± 0.24
Hexanal	Green grass, fat	20.03 ± 0.19	3.62 ± 0.23	nd
Benzeneacetaldehyde	Harsh, green, honey, cocoa	4.27 ± 0.36	6.95 ± 0.02	4.48 ± 0.05
Nonanal	Fat, citrus, green beany	0.2 ± 0.01	2.68 ± 0.03	1.97 ± 0.2
2-Pentyl-furan	Green beans, butter	12.7 ± 0.21	5.6 ± 0.31	5.16 ± 0.25
Acetophenone	Sweet, flower, almond	3.43 ± 0.35	6 ± 0.45	3.19 ± 0.13
Toluene	Pungent, caramel, fruity, solvent	3.78 ± 0.17	7.07 ± 0.37	4.51 ± 0.36
Octane	Green grass, fat, citrus, soap	nd	8.66 ± 0.45	8.4 ± 0.4
Limonene	Citrus, mint	10.13 ± 0.15	13.23 ± 0.1	58.29 ± 0.6

All data are the means and standard deviation of triplicate measurements. Abbreviations are as in Table 1

Green lentil flour had a limonene amount close to WF. In relatively high percentages octane, benzeneacetaldehyde, 1-hexanol, 2-pentyl-furan, acetophenone were also detected. The major volatile compounds identified from WF were hexanal, 1-hexanol and limonene. Except for limonene, hexanal and 1-hexanol were found in the smallest amounts in FLR and FLG (hexanal was not detected in red lentil flour). Alcohols are mostly formed from enzymatic oxidation (lipoxidase) of lipids. Physical damage, storage and processing of seeds could lead to the formation of alcohols [31, 32]. Volatile alcoholic compounds have distinct characteristics and they could therefore affect the taste and flavour of flours; for example 1-hexanol has an herbaceous, mild, sweet, green fruity odour and an aromatic flavour. Enzymatic or autoxidative decomposition of unsaturated fatty acids, mainly linoleic acid, as well tissue disruption, could lead to the formation of aldehydes in flours [32, 33]. The aldehydes identified may affect the taste and flavour perceived since they have different characteristics, e.g., hexanal has a fatty, green, grassy, fruity odour and taste, nonanal has green beany odour and taste. Also, aromatic compounds (such as toluene, 2-pentyl-furan), volatile alkanes, ketones are derived from oxidation of unsaturated fatty acids and could affect the characteristic aroma and taste of flours. Limonene, the main volatile compound found, belongs to terpenes class and is frequently found in essential oils; the presence of this compound could result from the degradation of carotenoids, thus the red lentil flour had the highest content in limonene (58.29%). Red lentil varieties have been reported with higher total carotenoids content than green varieties [6]. Limonene gives citrus and mind notes to flours flavour.

### Development and characterization of wheat-lentils composite flours with optimised folic acid content

Taking into account the high folic acid content of lentil flours coupled with the high consumption amount of cereal based products, it seems that cereal flours containing products may have a high perspective for ensuring the recommended daily intake of folic acid. Consequently, during this work it was aimed to develop and characterize different wheat-lentils composite flours with optimised folic acid content. For developing new composite flours, an average consumption of 250 g day^−1^ bread was taken (World Health Organization [12]), meaning 150 g day^−1^ of flour when using flour: bread technological ratio of 0.6. The water absorption coefficient for wheat: lentil flour blend was 62% (data not shown). Further, it was aimed that this amount of composite flour (150 g) consumed daily by an adult person, has to include the recommended folic acid daily intake of 200 μg [8]. This means a folic acid content of 1333.3 μg kg^−1^ of composite flour. In order to achieve this targeted folic acid content, two composite flours were developed by mixing wheat flour with either green or red lentils flours and taking into consideration the low folic acid content of wheat flour (106.2 µg kg^−1^) coupled with the high folic acid content of green (1683.6 µg kg^−1^) and red (2244.8 µg kg^−1^) lentils. The wheat (W) and lentil (L) flours percentages used for the composite, were calculated by using the total material balance (Eq. 1) and folic acid content material balance (Eq. 2):

1 $${\text{W }} + {\text{L }} = 100$$

2 $${\text{W}}\cdot{\text{fa}}_{\text{w}} + {\text{L}}\cdot{\text{fa}}_{\text{l}} = 100\cdot{\text{fa}}_{\text{t}}$$

where W is the amount of wheat flour, L is the amount of lentil flour, *fa*_*w*_ and *fa*_*l*_ are the folic acid content of wheat and lentil flour, respectively, while *fa*_*t*_ is the folic acid target content (i.e., 1333.3 µg kg^−1^) of the new composite flours developed. For the wheat–green lentil composite flour (W–GL) a percentage of 22.21% of wheat and 77.79% of green lentil were computed, while for the wheat–red lentil composite flour (W–RL) a percentage of 42.62% of wheat and 57.38% of red lentils flour, respectively. As compared to W–GL, a higher content of wheat flour is included in this composite while the folic acid content registered for red lentil flour was higher (2244.8 µg kg^−1^) as compared to the one obtained for green lentils (1683.6 µg kg^−1^). Further, for better describing the new flours developed, a prediction of their composition was based on previously computed percentages for each flour and biochemical component (Table 3). The new composite flours proposed by this work, showed improved characteristics as compared to the standard wheat flour. For example, both W–GL and W–RL roughly doubled the amount of crude protein (222 and 20.59 g kg^−1^ as compared to 11.7 g kg^−1^ for wheat) and showed higher contents for most important biochemical compounds, especially minerals (~ 2 times increased for Mn; ~ 4 times increased for Ca, Zn, Mg and ~ 5–6 times for Fe, Cu and K), lipids (doubled the amount of n − 3 fatty acids) and most important amino acids (especially for lysine content which showed an increase of ~ 17 times). However, even if the new wheat/lentils composite flours showed clearly improved nutritional characteristics, further studies are necessary for finding and optimising the bread technology and/or other flour based food containing these new developed composite flours, in order to obtain high quality products. Also, studies regarding the bioavailability of folates are needed since the bioavailability of natural folates appears to be lower compared to the administered form of folic acid [34].

**Table 3:** Wheat–green lentil (W–GL; 22.21–77.79%) and wheat–red lentil (W–RL; 42.62–57.38%) composite flours advanced biochemical characterization (prediction)

Chemical compounds	W–GL	W–RL
Folic acid (µg/100 g)	133.33	133.33
Minerals (mg/100 g DM)		
Calcium (Ca)	63.26	57.58
Iron (Fe)	6.12	5.38
Potassium (K)	765.33	648.41
Zinc (Zn)	3.91	2.88
Magnesium (Mg)	100.84	84.03
Manganese (Mn)	1.45	1.41
Copper (Cu)	1.06	0.79
Amino acids (g/16 g N)		
Lysine	4.50	3.48
Threonine	3.66	2.56
Valine	3.31	2.36
Isoleucine	2.51	1.92
Leucine	5.08	3.84
Phenylalanine	3.83	2.68
Arginine	5.72	4.60
Alanine	3.37	2.40
Proline	3.87	3.02
Glycine	3.32	2.36
Glutamic acid	12.21	9.94
Aspartic acid	9.92	7.18
Fatty acids (% of total fatty acids)		
10:00	0.01	0.01
12:00	0.02	0.04
14:00	0.41	0.30
15:00	0.12	0.14
Aza	0.04	0.06
16:00	16.99	16.17
16:1(n − 9)	0.09	0.14
16:1(n − 7)	0.18	0.10
17:00	0.20	0.15
17:1(n − 9)	0.08	0.08
18:00	2.23	1.50
18:1(n − 9)	20.35	22.68
18:1(n − 7)	0.89	0.78
18:2(n − 6)	47.87	47.71
18:3(n − 3)	8.36	7.91
20:00	0.48	0.40
20:1(n − 9)	0.50	0.69
20:2(n − 6)	0.06	0.08
21:00	0.09	0.08
22:00	0.44	0.42
22:1(n − 9)	0.10	0.12
24:0	0.30	0.29
24:1(n − 9)	0.03	0.04
ΣSFAs	21.43	19.63
ΣMUFAs	22.23	24.62
ΣPUFAs	56.30	55.70
n − 3ΣPUFAs	8.36	7.91
n − 6ΣPUFAs	47.95	47.79
n − 6/n − 3	6.74	8.05
PUFAs/SFAs	2.63	2.83
Total fat (%dw)	1.32	1.22

Abbreviations are as in Table 1

### Baking of wheat-lentils composite flours with optimised folic acid content

**Fig. 1:**
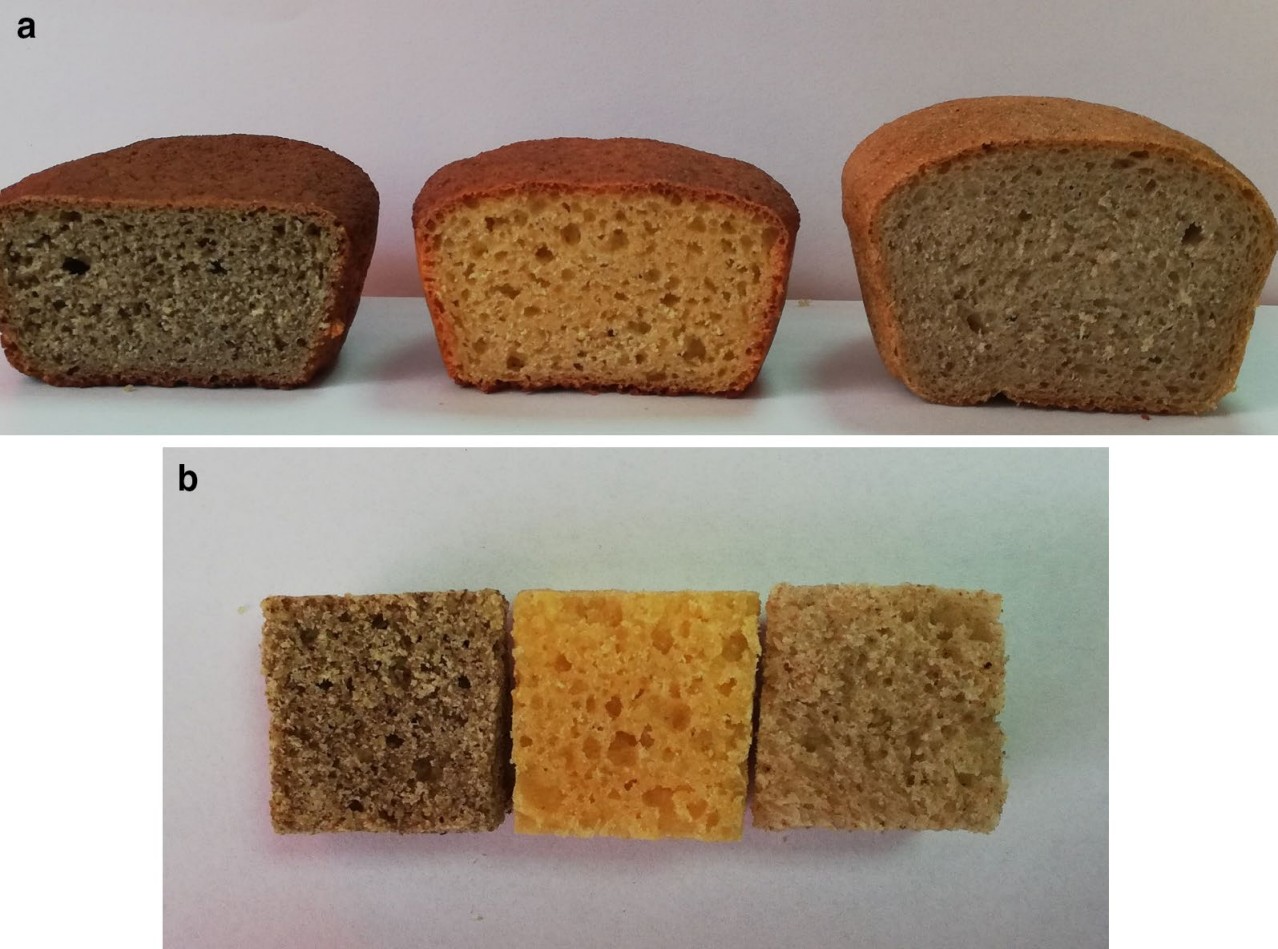
Sections (**a**) and middle crumb (**b**) of the obtained wheat-lentils bread samples as compared to a control wheat bread (from left to right: wheat–green lentil, wheat–red lentil and wheat control bread samples)

In order to assess the organoleptic and textural properties of the bread as a result of gluten content decreasing, while lentil flours were added, the baking behaviour of the new developed wheat-lentils composite flours with optimised folic acid content was tested. The Fig. 1a presents the sections of the obtained wheat-lentils bread samples, in comparison with wheat control bread. A good baking behaviour was observed for the wheat–red lentil bread, while for the wheat–green lentil composite flour, encouraging results were obtained. Texture profile analysis was considered the most relevant test in order to objectively compare the bread samples, the main textural parameters being summarized on Table 4. As expected the wheat control bread showed the lowest values of hardness, gumminess and chewiness, while having the highest springiness index (0.91) and cohesiveness (0.72). Among wheat–red lentil and wheat–green lentil composite flours, the former performed better when baked, showing a fivefold higher first cycle hardness than control, while having close values of springiness index (0.85) and cohesiveness (0.48) to wheat bread. The extensive green lentil addition (77.79%) for wheat–green lentil composite flour, caused a severe gluten reduction, which further explained the satisfactory texture parameters of wheat–green lentil bread samples. On the other hand, the moderate gluten reduction as a consequence of red lentil addition (57.38%) determined a wheat–red lentil bread with acceptable texture parameters, as compared to control wheat bread—increasing of first and second hardness values, gumminess and chewiness indexes, as well as the slight decrease of cohesiveness and springiness index, proving the baking capability of this nutritionally optimised composite flour.

**Table 4:** Texture profile analyses for bread samples obtained from wheat–lentil composite flours as compared to wheat control bread (means ± standard deviations)

Bread sample	Sample length [mm]	Hardness cycle 1 [g]	Total work cycle 1 [mJ]	Hardness cycle 2 [g]	Total work cycle 2 [mJ]	Cohesiveness [n.a.]	Springiness index [n.a.]	Gumminess [g]	Chewiness index [g]
Wheat Control Bread (B_W)	25.84 ± 0.34	547 ± 74	37.8 ± 5.9	512 ± 65	29.1 ± 4.3	0.72 ± 0.02	0.91 ± 0.01	395 ± 48	359 ± 45
Wheat–Green lentil bread (B_W–GL)	25.54 ± 0.36	3723 ± 775	224.4 ± 47.0	2830 ± 563	99.4 ± 22.4	0.39 ± 0.01	0.86 ± 0.13	1466 ± 335	1294 ± 499
Wheat–Red lentil bread (B_W–RL)	25.19 ± 1.03	2693 ± 188	168.4 ± 20.2	2186 ± 153	88.9 ± 7.2	0.48 ± 0.03	0.85 ± 0.01	1285 ± 105	1091 ± 77

## Conclusions

In this study two composite flours [wheat–green lentils (22.21–77.79%) and wheat–red lentils (42.62–57.38%)] with an optimised content of folic acid (1333.3 µg kg^−1^) to assure the recommended daily intake were developed. Also, these composite flours were advanced characterized in terms of minerals, amino acids, fatty acids and volatile compounds. Even if these composites have optimised nutritional properties, future studies are required in order to optimise the bakery products formulation and technological process for obtaining an enhanced folates bioavailability. Further studies will be conducted with sourdough, germination or selected strains of lactobacillus and yeasts in order to achieve a good folates daily intake from bakery products obtained from wheat-lentils composite flours.
